# A new solitary free-living species of the genus *Sphenopus* (Cnidaria, Anthozoa, Zoantharia, Sphenopidae) from Okinawa-jima Island, Japan

**DOI:** 10.3897/zookeys.606.9310

**Published:** 2016-07-21

**Authors:** Takuma Fujii, James Davis Reimer

**Affiliations:** 1 Research Center for the Pacific Islands Amami Station, Kagoshima University, Naze-Yanagimachi 2-1, Amami, Kagoshima 894-0032, Japan; 2 Graduate School of Engineering and Science, University of the Ryukyus, 1 Senbaru, Nishihara-cho, Okinawa 903-0213, Japan; 3 Molecular Invertebrate Systematics and Ecology Laboratory, Department of Biology, Chemistry & Marine Sciences, Faculty of Science, University of the Ryukyus, 1 Senbaru, Nishihara, Okinawa 903-0213, Japan; 4 Tropical Biosphere Research Center, University of the Ryukyus, 1 Senbaru, Nishihara, Okinawa 903-0213, Japan

**Keywords:** Zoantharia, Sphenopus, new species, free-living, enclosed bay, identification key

## Abstract

A new species of free-living solitary zoantharian is described from Okinawa, Japan. *Sphenopus exilis* **sp. n.** occurs on silty seafloors in Kin Bay and Oura Bay on the east coast of Okinawa-jima Island. *Sphenopus exilis* **sp. n.** is easily distinguished from other *Sphenopus* species by its small polyp size and slender shape, although there were relatively few differences between *Sphenopus exilis* **sp. n.** and *Sphenopus marsupialis* in the molecular phylogenetic analyses. Currently, very little is known about the ecology and diversity of *Sphenopus* species. Thus, reviewing each species carefully via combined morphological and molecular analyses by using newly obtained specimens from type localities is required to clearly understand and distinguish the species within the genus *Sphenopus*.

## Introduction

The suborder Brachycnemina (Cnidaria: Anthozoa: Hexacorallia: Zoantharia) consists of zoantharians commonly found in shallow warm waters, as almost all species within this group contain endosymbiotic photosynthetic *Symbiodinium* spp. (e.g. Swain 2010). The genus *Sphenopus* belongs to the family Sphenopidae within Brachycnemina based on its brachycnemic mesenterial arrangement, mesogleal sphincter muscles, and heavy encrustation of granules into the body column. This genus is unique as *Sphenopus* individuals consist of free-living solitary polyps, in contrast to not only other Sphenopidae and Brachycnemina species but also to all other known zoantharians. This unique feature is considered to be an adaptation to *Sphenopus*’ muddy/sandy sea floor habitats that are difficult for most sessile benthos to inhabit (e.g. Soong et al. 1999, Reimer et al. 2012). Here they risk burial, which would require them to shed sediments in order to survive as seen in free-living scleractinians (e.g. Schuhmacher 1977, Fisk 1982, Bongaerts et al. 2013, Sentoku et al. 2016).

Three species are currently considered valid within the genus *Sphenopus*; *Sphenopus marsupialis* (Gmelin, 1791), *Sphenopus arenaceus* Hertwig, 1882, and *Sphenopus pedunculatus* Hertwig, 1888. In contrast to *Sphenopus marsupialis* with a wide distribution in the Indo-Pacific (Soong et al. 1999, Reimer et al. 2012, 2014), there have been no further records of *Sphenopus arenaceus* and only one additional record of *Sphenopus pedunculatus* (in Reimer et al. 2014) after their original descriptions. Although *Sphenopus marsupialis* has been reported in some field guides, formal taxonomic studies based on specimens are limited (Soong et al. 1999, Reimer et al. 2012). Thus, comparatively very little is known about the species diversity of the genus *Sphenopus*.

Recently, we discovered comparatively small *Sphenopus* specimens (polyp lengths <2.5 cm) from the shallow silty seafloors of enclosed bays on the east coast of Okinawa-jima Island, Japan. Combined morphological and molecular phylogenetic analyses lead us to conclude that the specimens belong to a previously unknown species. Thus, in this paper, a new *Sphenopus* species is formally described and a dichotomous key to identify all known *Sphenopus* species is provided. This report represents only the second formal record of this genus in Japan after Reimer et al. (2016).

## Material and methods

**Sample collection.** Specimens from Okinawa were collected by SCUBA. Prior to collecting, *in situ* images of expanded polyps were taken to assist in morphological analyses (colour, tentacle counts and size, polyp form). Half of the specimens collected were preserved in 99% EtOH for DNA analyses, and the other specimens were fixed for morphological analyses in 5 to 10% formalin sea water after anesthesia using MgCl_2_, and subsequently transferred to 70% EtOH some days later.

**Figure 1.**
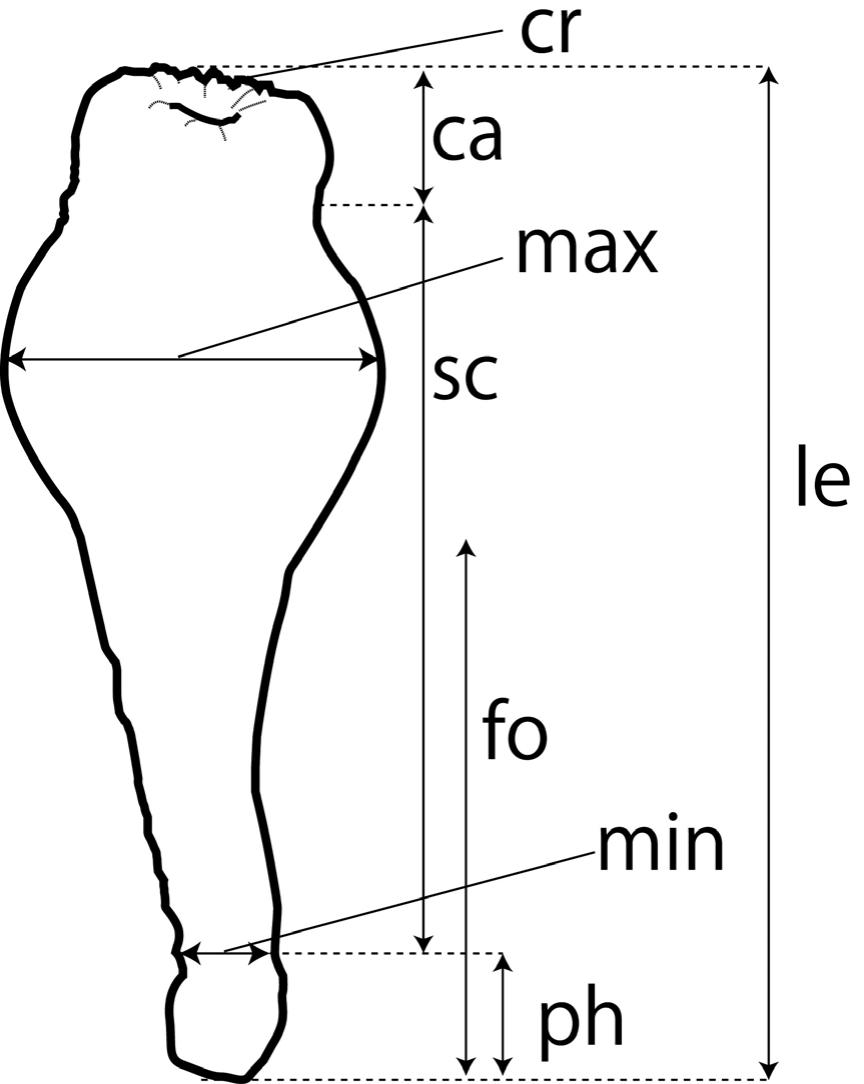
Diagram of external morphology of a contracted polyp of *Sphenopus exilis* sp. n.. ca = capitulum; cr = capitular ridge; fo = foot; le = length; max = maximum width; min = minimum width; ph = physa; sc = scapus, column.

**Morphological analyses.** The lengths, maximum widths (largest diameter of column) and minimum widths (width at the top of physa where the aboral ampullaccous ends) of the column of preserved polyps were measured using calipers to the nearest 0.1 mm (Figure 1). Gross shape of polyps, color of polyps, and numbers and lengths of the tentacles were recorded utilizing in situ images. Internal morphology was observed from horizontally and longitudinally hand-cutting polyps through the actinopharynx using a dissecting microscope.

**Cnidae.** Undischarged cnidae were measured from small pieces of tissue from the tentacles, column (external portion), actinopharynx, and mesenterial filaments of specimen NSMT-Co1576 (MISE-TF-107; fixed in 5~10% formalin seawater). Images of cnidae were obtained by differential interference contrast microscopy, and measured using the software ImageJ (National Institute of Health, Bethesda, Maryland, USA). Cnidae nomenclature generally followed England (1991) and Ryland and Lancaster (2003). However, both Schmidt (1974) and Hidaka et al. (1987, 1992) have suggested basitrichs and microbasic b-mastigophores are the same type of nematocyst, and in this study, as in recent zoantharians studies (e.g. Kise and Reimer 2016, Ryland and Ward 2016), these two types were treated as the same.

**DNA processing and amplification.** DNA was extracted from ethanol preserved specimens by following a guanidine extraction protocol (Sinniger et al. 2010). PCR amplifications were performed for mitochondrial cytochrome oxidase subunit I (COI), mitochondrial 16S ribosomal DNA (mt 16S rDNA), and the internal transcribed spacer region of ribosomal DNA (ITS-rDNA) region using the primer pairs HCO and LCO (Folmer et al. 1994), 16SarmL (modified primer for mt 16S rDNA used in Sinniger et al. 2008, see Fujii and Reimer 2011) and 16SBmoH (Sinniger et al. 2005), and ITSf and ITSr (Swain 2009), respectively. Amplified PCR products were sequenced in both directions by Fasmac (Atsugi, Kanagawa, Japan).

**Phylogenetic analyses.** New sequences obtained in this study were deposited in GenBank (accession numbers: COI, KX400760–KX400768; mt 16S rDNA, KX400756–KX400759; ITS-rDNA, KX400769–KX400772). Obtained DNA sequences were manually aligned using Bioedit ver. 7.1.3.0 (Hall 1999). The nucleotide sequences of mt 16S rDNA, COI, and the ITS-rDNA region from specimens were separately aligned with previously obtained Sphenopidae (*Palythoa* and *Sphenopus*) sequences deposited in GenBank. Some sequences that were too short in length were removed from the analyses. For outgroups, sequences of *Zoanthus sansibaricus* (suborder Brachycnemina, family Zoanthidae) were used for all three DNA alignments’ trees. Indels were kept unedited in the alignments of mt 16S rDNA. All phylogenetic alignments are available from the corresponding author.

For phylogenetic analyses of mt 16S rDNA, COI, and ITS-rDNA the same methods were independently applied. The maximum-likelihood (ML) method was performed using MEGA5 (Tamura et al. 2011), with 500 replicates performed using an input tree generated by BIONJ with the general time-reversible model (Rodriguez et al. 1990) of nucleotide substitution incorporating invariable sites and a discrete gamma distribution (eight categories) (GTR+I+C). The proportion of invariable sites, a discrete gamma distribution, and base frequencies of the model were estimated from the dataset. Bayesian trees for nuclear ITS-rDNA region were made by Mr. Bayes 3.2.5 (Ronquist and Huelsenbeck 2003) under GTR+I+C. One cold and three heated Markov chains Monte Carlo (MCMC) with default-chain temperatures were run for 10 million generations, sampling log-likelihoods (InLs), and trees at 100-generation intervals (100,000 InLs and trees were saved during MCMC). The likelihood plots for ITS-rDNA datasets suggested that MCMC reached the stationary phase after the first 10,000 generations (standard deviation of split frequencies = 0.004361). Thus, the remaining 90,000 trees of ITS-rDNA were used to obtain clade probabilities and branch-length estimates.

## Results

### Suborder Brachycnemina Haddon and Shackleton, 1891 Family Sphenopidae Hertwig, 1882 Genus *Sphenopus* Steenstrup, 1856

#### Sphenopus exilis sp. n.

Taxon classificationAnimaliaZoanthariaSphenopidae

http://zoobank.org/30C107C6-8104-4EC9-9DC9-CF2DEE4A9638

Figures 2 , 3

##### Holotype.

**Figure 2.**
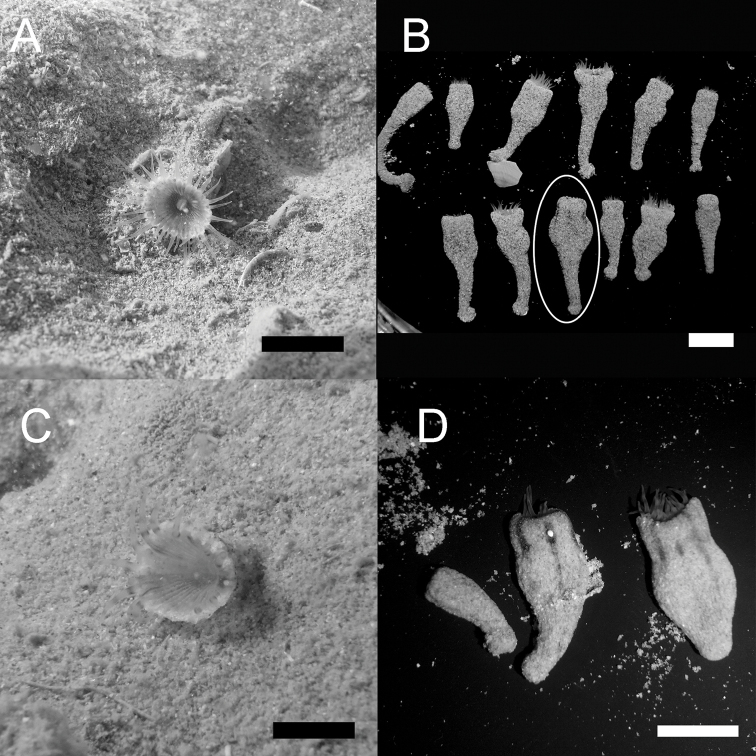
Polyps of *Sphenopus exilis* sp. n. **A** In situ image of *Sphenopus exilis* sp. n., polyp with no black patterns, from the type locality in Kin Bay, Okinawa, Japan on 29 October 2011 **B** Polyps of NSMT-Co1576 & NSMT-Co1577 from Kin Bay, Okinawa-jima Island, Japan. The white circle points to the holotype **C** In situ image of NSMT-Co1578 from Oura Bay, Okinawa-jima Island, Japan, on 13 November 2012. Faint black patterns and bands appear on the oral disc and the tentacles **D** Polyps of lot number NSMT-Co1578 showing phenotypic variation with black stripes on the upper part of the polyps. Scale bars: 1 cm.

Specimen number NSMT-Co1576 (MISE-TF-107): Kin Bay, Uruma, Okinawa-jima Island, Japan (26°22'25"N, 127°53'30"E), 15 m depth, collected by Takuma Fujii, 29 October 2011, fixed in 5–10% SW formalin, deposited in National Museum of Nature and Science, Tokyo, Japan (NSMT). Polyp length 2.4 cm, maximum width 0.8 cm, minimum width 0.3 cm. Figure 2B.

##### Paratypes.

Specimen number NSMT-Co1577 (MISE-TF-107), a lot of total 11 polyps collected on the same dive, collection data same as holotype, five polyps fixed in 5–10% formalin, six polyps fixed in 99% EtOH, polyp length 1.3 to 2.2 cm (average 1.7±0.3 cm), maximum width 0.4 to 1.0 cm (average 0.5±0.2 cm), minimum width 0.2 cm, deposited in NSMT. GenBank accession numbers: COI, KX400760–KX400768; mt 16S rDNA, KX400756–KX400759; ITS-rDNA, KX400769–KX400772. Figure 2A and B; Specimen number RMNH Coel. 42121 (MISE-TF-144): a lot of total 16 polyps collected on the same dive, Kin Bay, Uruma, Okinawa-jima Island, Japan (26°22'25"N, 127°53'30"E), 15 m depth, collected by Takuma Fujii, 24 May 2012, 11 polyps fixed in 5–10% formalin, five polyps fixed in 99% EtOH, polyp length 1.1 to 2.2 cm (average 1.7±0.4 cm), maximum width 0.4 to 0.5 cm (average 0.5±0.1 cm), minimum width 0.1 to 0.3 cm (average 0.2±0.1), deposited in Naturalis Biodiversity Center, Leiden, Netherlands (RMNH); Specimen number NSMT-Co1578 (MISE-TF-151), a lot of total six polyps collected on the same dive, Oura Bay, Nago, Okinawa-jima Island, Japan (26°32'29"N, 128°3'16"E), 17 m depth, collected by Takuma Fujii, 13 November 2012, five polyps fixed in 5–10% formalin, 1 polyp fixed in 99% EtOH, polyp length 1.0 to 1.9 cm (average 1.5±0.4 cm), maximum width 0.3 to 1.1 cm (average 0.7±0.3 cm), minimum width 0.1 to 0.3 cm (average 0.2±0.1 cm), deposited in NSMT. Figure 2C and D.

##### Diagnosis: external morphology.

Solitary, cylindrical polyp. Length of polyps 1.0 to 2.4 cm (average 1.7±0.3 cm), maximum width 0.3 to 1.1 cm (average 0.6±0.2 cm), minimum width 0.1 to 0.3 cm (average 0.2±0.1 cm) (n=34). Tentacles longer than half diameter of the expanded oral disc (Figure 2A). Oral disc gently hollowing into mouth, with stellate grooves as many as tentacles (Figure 2A, C). Capitular ridges present but not strongly pronounced when polyps closed (Figure 1). The upper part of the polyp between capitulum and the column slightly constricted (the width of the most constricted region approximately 0.1 cm to 0.4 cm thinner than the width of contracted capitulum) when polyp contracted (Figures 1, 2B, D). Upper part of the column generally thick and oval (Figures 1, 2B, D). Aboral narrow bottom portion of column extended (=foot), thinner than upper portion of column, like a cone (Figures 1, 2B, D), with the distal portion round and thicker than the extended foot (=physa) (Figures 1, 2B, D). Column smooth, with encrusted fine dense sand particles. Occasionally broken piece(s) of bivalve shells attached to the aboral end (Figure 2B).

##### Diagnosis: internal morphology.

**Figure 3.**
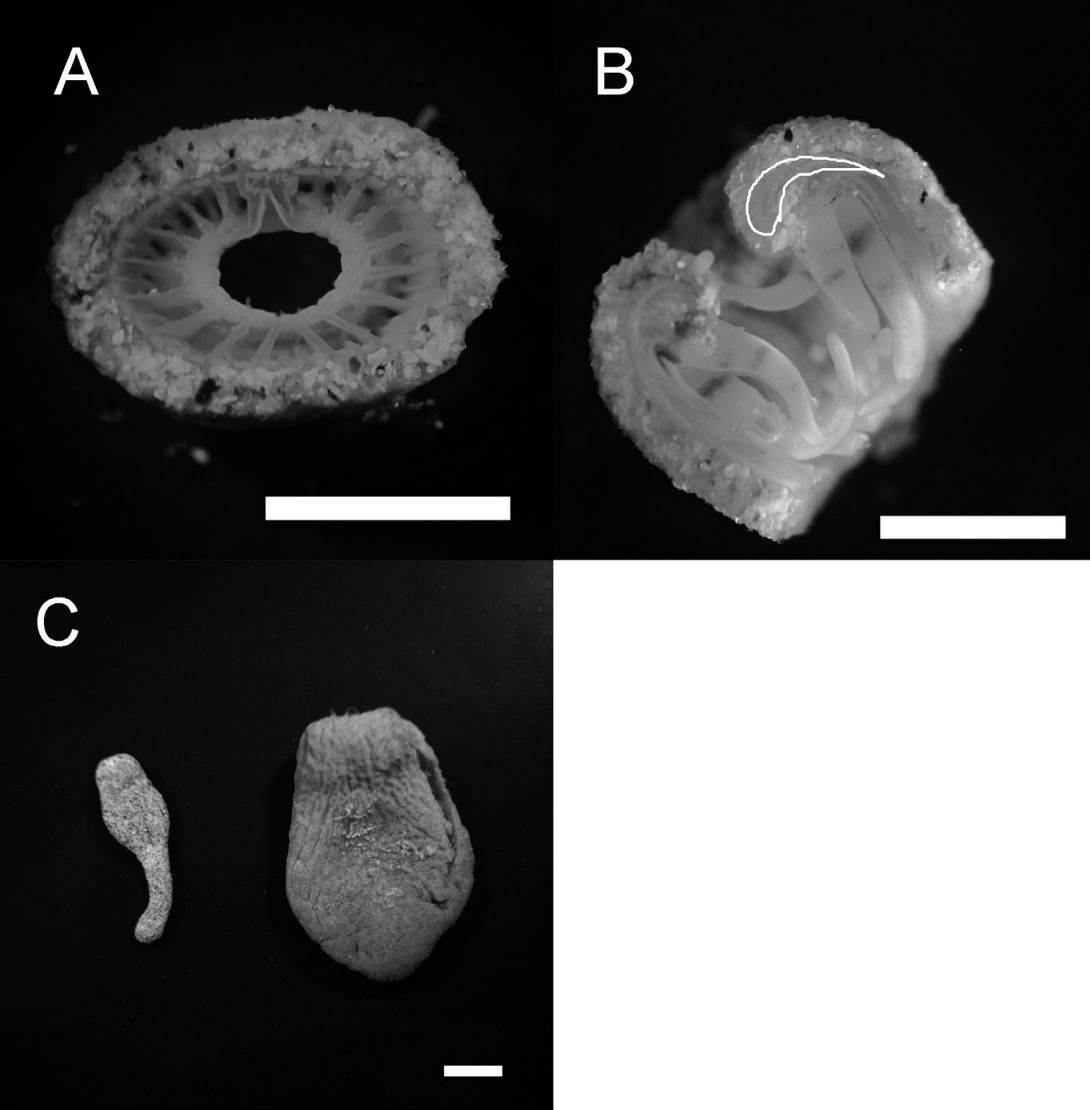
Morphological features of *Sphenopus exilis* sp. n. **A** Cross section of holotype NSMT-Co1576 through the actinopharynx showing the mesenterial arrangement and dense sand encrustations **B** Well-developed mesogleal sphincter muscles visible on a hand-cut longitudinal section of the holotype NSMT-Co1576 **C** Comparison of polyp shape between *Sphenopus exilis* sp. n. NSMT-Co1577 and *Sphenopus marsupialis* (from Brunei, refer to Reimer et al. 2012).

Fine sand particles heavily encrusted into ectoderm and mesoglea. Mesenteries in brachycnemic arrangement. Mesentery number 36, complete 18, incomplete 18 (Figure 3A; n=6 polyps). Single siphonoglyph apparent. Mesogleal sphincter muscle well developed, visible under dissecting microscope (Figure 3B). Endosymbiotic *Symbiodinium* spp. (zooxanthellae) absent (=azooxanthellate).

##### Diagnosis: cnidae.

Basitrichs and spirocysts in tentacles and actinopharynx. Basitrichs, holotrichs, microbasic p-mastigophores and basitrichs in mesenterial filaments. Holotrichs in column (Table 1).

**Table 1.** Cnidae types and sizes in different tissue sections of the holotype of *Sphenopus exilis* sp. n.

		Image (Scale bars: 50 μm)	Length*	Width*	Frequency**
Tentacle	Basitrich	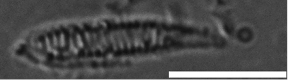	10.4 (21.4–19.1)	3.0 (2.6–3.3)	Occasional (n=8)
	Spirocyst	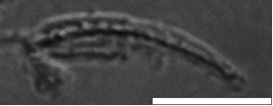	13.1 (12.0–14.5)	2.6 (2.4–2.8)	Numerous (n=20)
Column	Holotrich	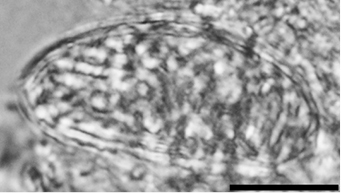	26.7 (16.3–34.5)	14.7 (12.5–16.2)	Rare (n=3)
Actinopharynx	Holotrich	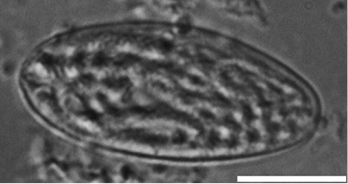	28.0 (21.0–34.9)	10.5 (5.5–15.5)	Rare (n=2)
	Basitrich	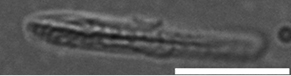	26.9 (26.4–27.4)	4.4 (3.9–5.0)	Numerous (n=20)
	Spirocyst	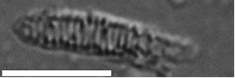	13.4 (10.8–15.2)	2.9 (2.8–2.9)	Common (n=13)
Filaments	Holotrich	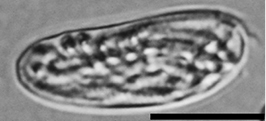	21.4 (17.0–25.6)	10.4 (5.9–15.25)	Occasional (n=4)
	Basitrich	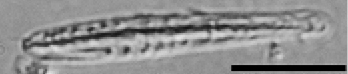	31.1 (30.6–31.6)	3.2 (2.8–3.5)	Numerous (n=20)
	*p*-mastigophore	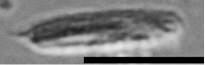	15.4 (14.6–16.2)	4.4 (3.7–4.9)	Occasional (n=5)

* Length and width: average, minimum–maximum, all sizes in µm.

** Frequency: n=number of examined cnidae in these analyses. Frequency in decreasing order; numerous, common, occasional, rare.

##### Habitat.

Specimens were found at approximately 10 to 20 m depths on the slopes of silty seafloors in enclosed bays. Most polyps semi-burrowed in silt, with only the open oral disc visible and protruding out from the seafloor.

##### Colour.

Tentacles and oral disc whitish and translucent in life. Faint black narrow horizontal bands appear on tentacles, and similar faint patterns on the oral disc of a few polyps (Figure 2C). Column colour of encrusted sand particles, a few polyps with 2 to 6 faint black vertical stripes approximately 15 mm wide on the upper part of the column, reaching from oral end to aboral end (Figure 2C, D).

##### Etymology.

Named from latin ‘exilis’ meaning ‘slender’ or ‘small’, as polyps have an elongate and narrow foot more slender than other known species in this genus to the exception of *Sphenopus pedunculatus*. Polyps of this species are also much smaller than those of all three other species in the genus.

##### Common name.

Hime-daruma-sunaginchaku (new Japanese name)

##### Molecular phylogeny.

The results of the phylogenetic analyses of both mitochondrial cytochrome oxidase subunit I(COI) and 16S rDNA showed very few differences between sequences of our specimens and those of *Sphenopus marsupialis*, as well as compared with those of various *Palythoa* species. These results are not incongruous with previous studies on the molecular phylogeny of family Sphenopidae, where intra-family variation levels of mitochondrial DNA sequences were relatively low (Reimer et al. 2006, 2012).

**Figure 4.**
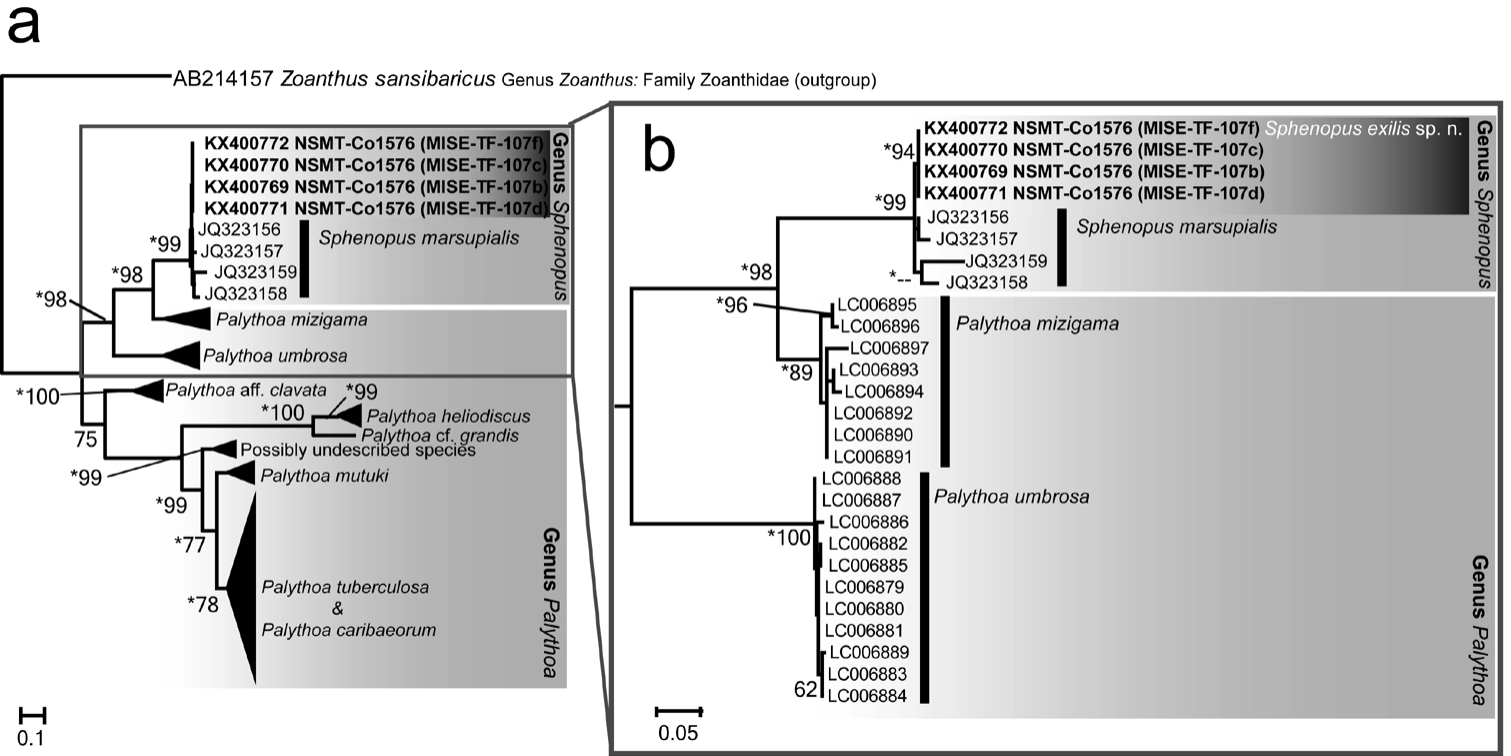
Maximum likelihood tree of nuclear internal transcribed spacer of ribosomal DNA (ITS-rDNA) region for newly obtained sequences from *Sphenopus exilis* sp. n. in this study along with previously published GenBank sequences of family Sphenopidae. Bootstrap values of ML >60% are shown at respective nodes. Nodes supported by Bayesian posterior probabilities >0.90 are marked with asterisks. Species names’ of sequences obtained from GenBank follow with accession numbers. The subtree shown in **b**) shows only the clade formed by genus *Sphenopus*, *Palythoa mizigama* and *Palythoa umbrosa*, delineated by the gray square in **a**).

The results of the phylogenetic analyses of nuclear internal transcribed spacer rDNA region showed *Sphenopus exilis* sp. n. forming a well-supported clade in the maximum likelihood and Bayesian analyses (Figure 4; ML=94%, Bayes=0.91). As well, together with sequences of *Sphenopus marsupialis*, *Sphenopus exilis* sp. n. formed a strongly supported *Sphenopus* clade (Figure 4; ML=99%, Bayes=1.00). In comparing the ITS-rDNA sequences between *Sphenopus exilis* sp. n. and *Sphenopus marsupialis*, there were 12 to 27 b.p. differences over a total 470 b.p. (=2.5~5.7% difference).

##### Remarks.

Until now three species have been considered valid within *Sphenopus*; *Sphenopus marsupialis* (Gmelin, 1791), *Sphenopus arenaceus* Hertwig, 1882, and *Sphenopus pedunculatus* Hertwig, 1888. *Sphenopus exilis* sp. n. is easily distinguished from these other species by its small polyp size (length of *Sphenopus exilis* sp. n. <2.5 cm and width <1 cm), and by the shape of its elongated foot and physa. Polyps of both *Sphenopus marsupialis* and *Sphenopus arenaceus* are round on the aboral end, and not elongated as in *Sphenopus exilis* sp. n. (Figure 3C). Soong et al. (1999) examined various sized *Sphenopus marsupialis* collected from around Taiwan including small polyps without any narrow elongated foot (length < 2 cm). Additionally, Reimer et al. (2016) recently reported on a *Sphenopus marsupialis* specimen of the typical rounded shape and large size (~9 cm in height) from Okinawa-jima Island. No polyps with intermediate morphology between *Sphenopus marsupialis* and *Sphenopus exilis* sp. n. have ever been found. Thus, the specimens collected in this study cannot be considered to be immature polyps of *Sphenopus marsupialis*. The morphologically most similar species to *Sphenopus exilis* sp. n. is *Sphenopus pedunculatus* as it also has a narrow foot, but *Sphenopus pedunculatus* is much larger than *Sphenopus exilis* sp. n., with polyp lengths of 2.4 to 3.2 cm and widths of 2 to 2.4 cm, and with approximately 60 mesenteries. As well, the aboral end of *Sphenopus pedunculatus* is shaped like a clasping disc, different from that of *Sphenopus exilis* sp. n. with a narrow rounded shape (Hertwig 1888, Reimer et al. 2014).

In contrast to the morphological differentiation from other *Sphenopus* species, only a few differences were found in molecular analyses. The COI sequences of *Sphenopus exilis* sp. n. were identical to those of *Sphenopus marsupialis*, *Palythoa tuberculosa* (Esper, 1805), and *Palythoa umbrosa* Irei, Singer & Reimer, 2015. However, it is known that the evolutionary rate of mitochondrial DNA markers is quite slow in most Anthozoa (Shearer et al. 2004; Huang et al. 2008; Stampar et al. 2014), and the nuclear ITS-rDNA region is currently the fastest evolving DNA marker that has been utilized for species-level analyses of suborder Brachycnemina (Reimer et al. 2007). Although there are only relatively few differences between the ITS-rDNA sequences of *Sphenopus exilis* sp. n. and *Sphenopus marsupialis* (2.5~5.7% sequence divergence), the formation of a supported monophyletic clade confirms the results of our morphological analyses that the specimens collected in this study belong to a species different from *Sphenopus marsupialis* (Figure 4). Moreover, these results suggest the possibility of the presence of multiple, cryptic species within *Sphenopus marsupialis* as previously mentioned by Soong et al. (1999).

Currently, very little is known about the ecology and species diversity of the genus *Sphenopus*, demonstrated by the fact that there have been no or few records of both *Sphenopus arenaceus* and *Sphenopus pedunculatus* within the last 100 years. Thus, morphological and molecular analyses of newly obtained specimens from type localities followed by reviewing each species’ description carefully are required to clearly understand the species distinction of *Sphenopus* species. As mentioned in previous studies, the phylogenetic results of this study indicate a need to re-examine the validity of the genus *Sphenopus* as it is positioned within the genus *Palythoa*, and by extension the definition of genera within the family Sphenopidae should be reconsidered (Reimer et al. 2012, Irei et al. 2015).

In the ITS-rDNA molecular phylogeny, it is notable that two recently described azooxanthellate *Palythoa* species from caves, *Palythoa umbrosa* and *Palythoa mizigama*, form a well-supported subclade with *Sphenopus exilis* sp. n. and *Sphenopus marsupialis*. As the phylogenetic relationship between *Sphenopus* and *Palythoa* is not yet clear, and likely does not reflect the traditional taxonomy (Reimer et al. 2012), construction of a large ITS-rDNA phylogeny with additional sequences from other *Palythoa* and *Sphenopus* species is needed. At the same time, investigation with additional DNA markers asides from the mt DNA and ITS-rDNA currently utilized in zoantharian phylogeny may be helpful.

### Key to species of genus *Sphenopus*

**Table:** 

1	Aboral end rounded, column shape oval, never having a very narrow stalk	**2**
–	Aboral end elongated and narrow, forming a foot	**3**
2	Polyp colored earthy gray	***Sphenopus marsupialis***
–	Polyp colored rusty red	***Sphenopus arenaceus***
3	Aboral end forms clasping disc, or the narrow stalk part of younger polyps very short compared to the oval part of the column. Comparatively large polyps (=polyp lengths > 2.4 cm, width > 2 cm) with approximately 60 mesenteries	***Sphenopus pedunculatus***
–	Aboral end forms a rounded anchor. Comparatively small polyps (length < 2.4 cm, width < 2 cm), and approximately 36 mesenteries	***Sphenopus exilis* sp. n.**

## Discussion

Unlike some other recently described zoantharian taxa such as Nanozoanthidae and Microzoanthidae, *Sphenopus exilis* sp. n. is not very small in size and does not inhabit a cryptic habitat. However, silty, sandy and rubble habitats are often overlooked in biodiversity surveys in favor of coral reef habitats, and this sampling bias has resulted in a relative lack of understanding of the diversity of these habitats and the evolutionary position of their inhabitants (Sheppard 1981, Obuchi et al. 2010, Fujii and Reimer 2011, Hoeksema 2012). In addition, not only are silty and muddy habitats less understood, but their ecosystem service value is often underestimated as well. For example, silty and muddy ecosystems on Okinawa-jima Island have been degraded by landfill dredging, and other development (Coral Reefs of Japan 2004), and over 35% of muddy tidal flats and shallow waters in Kin Bay have been lost (Uruma City Cultural Sea Museum 2007). Currently, *Sphenopus exilis* sp. n. is only known from two bays on the east coast of Okinawa-jima Island. Kin Bay, the type locality of *Sphenopus exilis* sp. n., has undergone ecological degradation over the past approximately 40 years (Reimer et al. 2015). The other locality, Oura Bay, is currently a center of controversy over proposed landfill and military base construction, while it is also known to house a unique benthic community (Fujii et al. 2015).

Thus, not only *Sphenopus exilis* sp. n., but also the diverse and various organisms that exclusively inhabit soft substrates in coral reef regions in the world face issues of decreasing habitat despite our lack of knowledge of their biodiversity (e.g. Cnidaria: Ceriantharia: Spier et al. 2012; Annelida: Polychaeta: Godet et al. 2008). *Sphenopus exilis* sp. n. serves as a clear reminder of how little we know of these ecosystems. It also provides a clear reason for better conservation and more exploration of the remaining silty and muddy areas.

## Supplementary Material

XML Treatment for Sphenopus exilis
